# Inducible T-Cell Co-Stimulator Impacts Chronic Graft-Versus-Host Disease by Regulating Both Pathogenic and Regulatory T Cells

**DOI:** 10.3389/fimmu.2018.01461

**Published:** 2018-06-22

**Authors:** Mengmeng Zhang, Yongxia Wu, David Bastian, Supinya Iamsawat, Jinsam Chang, Anusara Daenthanasanmak, Hung D. Nguyen, Steven Schutt, Min Dai, Fangping Chen, Woong-Kyung Suh, Xue-Zhong Yu

**Affiliations:** ^1^Department of Hematology, Xiangya Hospital, Central South University, Changsha, China; ^2^Department of Microbiology and Immunology, Hollings Cancer Center, Medical University of South Carolina, Charleston, SC, United States; ^3^Institut de Recherches Cliniques de Montréal (IRCM), Montreal, QC, Canada; ^4^Department of Hematology, Nanfang Hospital, Southern Medical University, Guangzhou, China; ^5^Department of Medicine, Medical University of South Carolina, Charleston, SC, United States

**Keywords:** inducible T-cell co-stimulator, chronic graft-versus-host disease, regulatory T cells, T follicular helper, follicular regulatory T

## Abstract

The incidence of chronic graft-versus-host disease (cGVHD) is on the rise and still the major cause of morbidity and mortality among patients after allogeneic hematopoietic stem cell transplantation (HCT). Both donor T and B cells contribute to the pathogenesis of cGVHD. Inducible T-cell co-stimulator (ICOS), a potent co-stimulatory receptor, plays a key role in T-cell activation and differentiation. Yet, how ICOS regulates the development of cGVHD is not well understood. Here, we investigated the role of ICOS in cGVHD pathogenesis using mice with germline or regulatory T cell (Treg)-specific ICOS deficiency. The recipients of ICOS^−/−^ donor grafts had reduced cGVHD compared with wild-type controls. In recipients of ICOS^−/−^ donor grafts, we observed significant reductions in donor T follicular helper (Tfh), Th17, germinal center B-cell, and plasma cell differentiation, coupled with lower antibody production. Interestingly, Tregs, including follicular regulatory T (Tfr) cells, were also impaired in the absence of ICOS. Using ICOS conditional knockout specific for Foxp3^+^ cells, we found that ICOS was indispensable for optimal survival and homeostasis of induced Tregs during cGVHD. Furthermore, administration of anti-ICOS alleviated cGVHD severity *via* suppressing T effector cells without affecting Treg generation. Taken together, ICOS promotes T- and B-cell activation and differentiation, which can promote cGVHD development; however, ICOS is critical for the survival and homeostasis of iTregs, which can suppress cGVHD. Hence, ICOS balances the development of cGVHD and could offer a potential target after allo-HCT in the clinic.

## Introduction

The morbidity and mortality associated with chronic graft-versus-host disease (cGVHD) has raised in the past two decades, due to improvements in patient care during the acute phase after allogeneic hematopoietic stem cell transplantation (allo-HCT), the use of peripheral blood stem cells instead of the bone marrow as grafts, and increasing age of donors or recipients (1–3). Patients with cGVHD show various clinical symptoms that can resemble those observed in various autoimmune disorders, such as fibrosis that can result in organ failure (4). The development of cGVHD involves aberrant effector T (Teff) and B cell activation, differentiation and costimulation, coupled with decreased regulatory T cell (Treg) generation and development (5–7).

Naïve CD4 T cells can differentiate into Th1, Th2, and Th17 subsets, among others. Tregs, *via* the transcription factor-Foxp3, limit the Teff and B cell response. IFN-γ, a Th1-signature cytokine, increases in patients in early stages post allo-HCT (3–8 months), but is notably decreased in later stages (≥9 months), suggesting that Th1 is required for the initiation of cGVHD (8–10). Th2 cells were originally reported as the dominate subset mediating cGVHD, yet conflicting data have obscured this finding (10–12). Th17 cells secrete IL-17 and IL-21 and can induce fibrosis (11–13). Thymic damage after conditioning leads to decreased Treg development, and subsequently an inability to suppress autoreactive and alloreactive immune cells (9, 14). T follicular helper (Tfh) cells provide support to B cells in germinal center (GC) formation, which facilitate B cell differentiation into plasma cells, leading to auto- and/or allo-antibody deposition in target organs (15). Follicular regulatory T (Tfr) cells, derived from natural Treg precursors, can control GC responses by suppressing B and Tfh cell responses (16). Thus, the aforementioned mechanisms contribute to both the complexity and development of cGVHD.

Inducible T-cell co-stimulator (ICOS), a member of the CD28 family, is expressed on activated murine T cells, NKT cells, and type 2 innate lymphoid cells. ICOS is implicated in almost all T-cell differentiation and cytokine production patterns (17). Depending on the context, ICOS has been documented to promote Th1 or Th2 skewing (18), maintain Th17 under inflammatory conditions (19–21), and promote Tfh cell differentiation (22, 23). ICOS also contributes to Treg development and suppressive function in both mice and humans; ICOS^−/−^ mice have reduced Treg percentage and number versus healthy controls (24–26). In addition, ICOS is important for GC formation and T-cell-dependent antibody responses, reflected by a profound defect in B-cell maturation and immunoglobulin isotype switching in both ICOS^−/−^ mice and humans associated with reduced help from Tfh cells (27–29).

Previous studies have shown that ICOS^−/−^ T cells have reduced IFN-γ yet elevated IL-4, which resulted in alleviated acute GVHD (aGVHD) (30); blocking ICOS confirmed this reduced GVHD severity (31). Antibody blockade of ICOS in mice with cGVHD using a bronchiolitis obliterans cGVHD mode can also improve pulmonary function by decreasing Tfh and GC responses (32). However, the role of ICOS in T-cell differentiation and Treg generation, development, and function is unknown in cGVHD. Utilizing a murine model of allogeneic bone marrow transplantation (BMT), we demonstrate a vital role for ICOS in promoting pathogenic T/B-cell differentiation, and further identified that ICOS was indispensable for Treg development and survival during cGVHD development. Importantly, we observe that ICOS blockade prior to cGVHD onset preserved Tregs and was efficacious in reducing cGVHD severity.

## Materials and Methods

### Mice

Wild-type (WT) C57BL/6 (B6, H-2K^b^, CD45.2), B6 Ly5.2 (CD45.1), and BALB/c (H-2K^d^) mice were purchased from National Cancer Institute (Frederick, MD, USA). Rag1^−/−^ B6 mice were purchased from The Jackson Laboratory (Bar Harbor, ME, USA). ICOS germline knockout (KO) (29) and ICOS^fl/fl^ (33) mice were generated in 129 background and backcrossed 12 generations into B6. ICOS^fl/fl^ mice were bred with Foxp3^YFP-Cre^ (JAX016959) mice to generate Treg-specific ICOS KO mice (Foxp3^YFP-Cre^ICOS^fl/fl^). Mice between 8 and 10 weeks old were used as recipients, and 6 and 8 weeks old mice were used as donors in this study. All mice were bred under specific pathogen-free conditions in the animal facility of the Medical University of South Carolina (Charleston, SC, USA). All animal experiments were approved by the Institutional Animal Care and Use of Committee.

### cGVHD Model

A major histocompatibility complex-mismatched (B6 to BALB/c) mouse model was used as previously described (34). Briefly, BALB/c recipients were lethally irradiated with total body irradiation (TBI) at 650 cGy using a RAD 320 X-ray Irradiator (Precision X-ray Inc., North Branford, CT, USA) and received 5 × 10^6^ T-cell-depleted bone marrow (TCD-BM) cells, with or without 0.5 × 10^6^ whole splenocytes (SPLs) or 0.25 × 10^6^ CD25-depleted splenocytes (CD25^−^SPLs) from WT, ICOS KO, Cre^−^ICOS^fl/fl^, or Foxp3^YFP-Cre^ICOS^fl/fl^ B6 donor mice *via* tail vein. Recipients were monitored for survival, body weight, and clinical syndromes of cGVHD described previously (35). As published previously, anti-ICOS (7E.17G9. G1, rIgG2b; produced at National Cell Culture, Minneapolis, MN, USA) or irrelevant rat-IgG were injected i.p. at 200 μg/mouse from day 0 to day 28, 3 times/week after BMT (31).

### aGVHD Model

BALB/c recipients were lethally irradiated with TBI at 700 cGy and injected with 5 × 10^6^ BM from Rag1^−/−^ B6 mice and enriched 0.5 × 10^6^ CD25^hi^Ly5.1^−^CD4 T cells on day 0, and then recipients were transferred with 0.5 × 10^6^ CD25^−^Ly5.1^+^ T cells on day 3. Recipients were monitored with survival, body weight loss, and clinical twice per week for 80 days.

### Flow Cytometry

Recipient’s splenocytes and thymocytes were isolated and stained for surface markers and intracellular markers and cytokines using standard flow cytometric protocols as previously described (35). Stained cells were analyzed by LSR II (BD Biosciences, San Jose, CA, USA) and Flow Jo (Tree Star, Ashland, OR, USA).

### Serum Autoantibody Detection

Serum autoantibodies were detected as previously described (35). Succinctly, double-stranded DNA (dsDNA) made from calf thymus were pre-coated on ELISA plate (Corning Inc.) and then incubated with diluted serum. Biotin-IgG, IgG1, and IgG2c (Southern Biotech) followed by HRP-streptavidin antibodies and TMB substrate (eBioscience) were utilized. Plates were read out by a Multiscan FC (Thermo Scientific, MA, USA) ELISA plate reader.

### Trichrome Staining

Six-micrometer cryosections were stained with a Masson trichrome staining kit (Sigma-Aldrich) for detection of collagen deposition. Collagen deposition was quantified on trichrome-stained sections as a ratio of area of blue staining to area of total staining by use of ImageJ 1.51s (National Institutes of Health, USA) analysis tool.

### iTregs Generation and Enrichment

CD4^+^CD25^−^ T cells were purified from WT or ICOS^−/−^ B6 spleens and lymph nodes by MACS. CD11c^+^ dendritic cells were purified from BALB/c mice using CD11c microbeads (Miltenyi). CD4^+^CD25^−^ T cells were co-cultured with CD11c^+^ DCs at 1:10 (DC:T cell) ratio with IL-2 (5 ng/ml), TGF-β (5 µg/ml), and retinoic acid (40 nM) for 5 days. iTregs were enriched from bulk culture using positive selection with CD25 microbeads and LS columns (Miltenyi). Purity of iTregs was usually 90–95% as in these experiments.

### Statistical Analysis

Results were presented as mean ± 1 SEM, a two-tailed Student’s *t*-test is utilized for accessing statistical significance among groups, and the log-rank test is utilized for evaluating recipient survival among groups by GraphPad Prism 6.

## Results

### ICOS Contributes to the Progression of cGVHD

**Figure 1:**
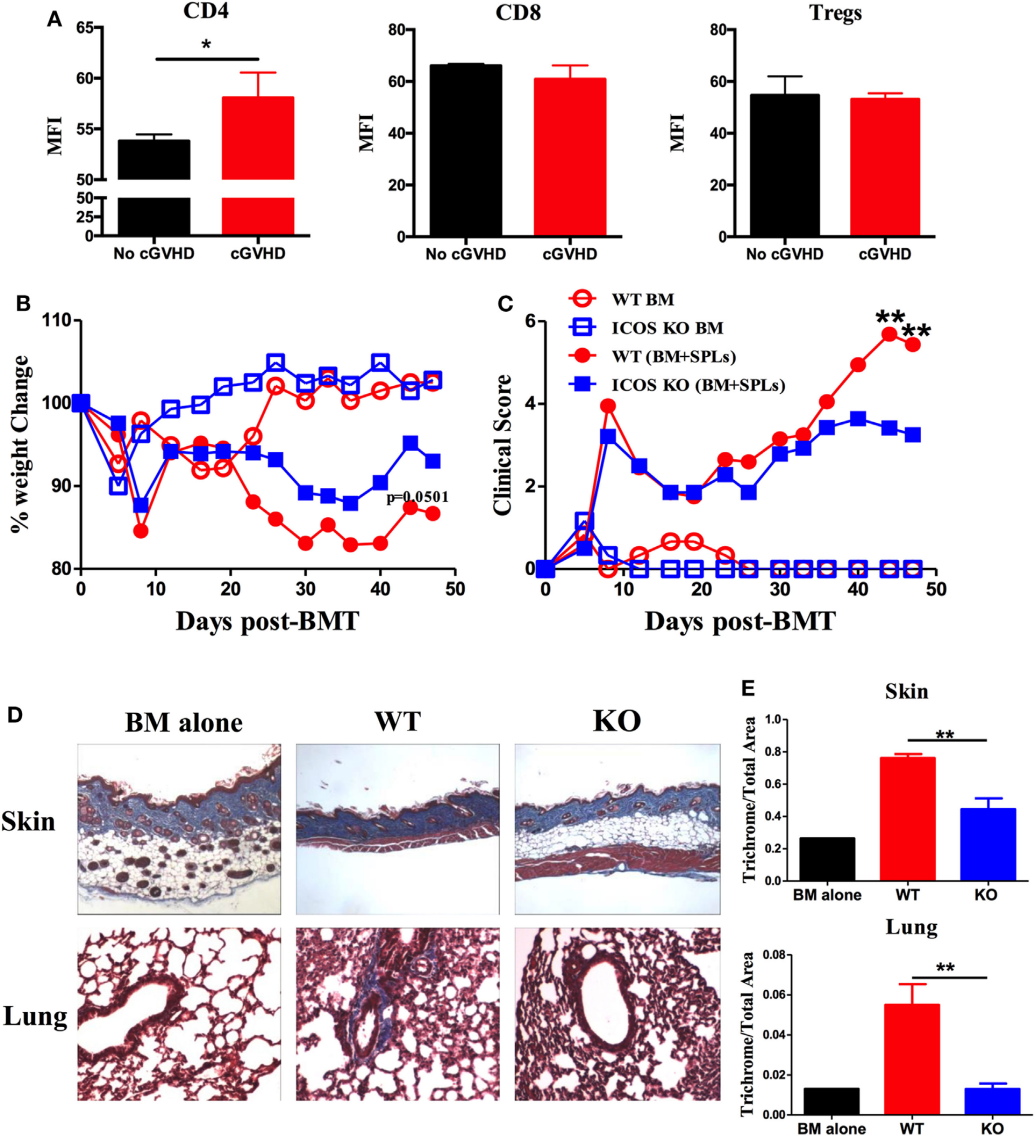
Inducible T-cell co-stimulator (ICOS) contributes to the progression of chronic graft-versus-host disease (cGVHD). **(A)** Lethally irradiated BALB/c mice were transplanted with 5 × 10^6^ T-cell-depleted bone marrow (TCD-BM) or plus 0.5 × 10^6^ whole SPLs from wild-type (WT) B6 mice. Spleens were processed and analyzed by flow cytometry. Mean fluorescence intensity (MFI) of ICOS on gated donor CD4, CD8, and regulatory T cells (Tregs) are shown, *n* = 3–5 mice/group. BALB/c mice were lethally irradiated and transferred with 0.25 × 10^6^ CD25^−^SPLs and 5 × 10^6^ TCD-BM from WT or ICOS^−/−^ mice on B6 background. Body weight **(B)** and clinical scores **(C)** of cGVHD were monitored bi-weekly for 50 days, *n* = 8 mice/group. Recipient skin and lung were harvested at day 60 after bone marrow transplantation (BMT) and processed for Masson’s trichrome staining. Representative images from one experiment are shown **(D)**. **(E)** Collagen deposition of skin and lung was qualified by ImageJ as the ratio of collagen area to the whole area of tissue, *n* = 4 mice/group. *p < 0.05 and **p < 0.01.

Inducible T-cell co-stimulator is expressed on activated CD4 and CD8 T cells and promotes T cell alloresponses to mediate GVHD (36). We therefore evaluated the ICOS expression on allogeneic T cells using a cGVHD transition model, B6 to BALB/c. We found that ICOS expression was significantly increased on donor CD4 T cells in the spleen of the recipients with cGVHD 60 days post-BMT compared with those without cGVHD; although ICOS expression was comparable on donor CD8 T cells and Tregs (Figure 1A). These data implicate ICOS expression on T cells in cGVHD, especially on the CD4 subset.

To test how ICOS affects the development of cGVHD, we initially used ICOS germline KO strain on B6 background as donors and BALB/c as recipients, in which the recipients develop aGVHD and transit to cGVHD (34). Consistent with previous reports (25, 28, 29, 37, 38), WT and ICOS KO displayed a comparable frequency of B220, CD4, and CD8, with a moderate decrease in regulatory and effector-memory CD4 T (Tem) cells (data not shown). Given that donor Tem have not been shown to impact GVHD development (39), and Tregs alleviate GVHD (40), we used CD25-depleted splenocytes plus TCD-BM as donor grafts to induce cGVHD. Recipients transplanted with ICOS^−/−^ grafts had reduced body weight loss (Figure 1B) and lower cGVHD clinical scores at later time points, but not at early time points compared with those receiving WT grafts (Figure 1C). As fibrosis is a key feature of cGVHD (6), we measured fibrosis in the recipient skin and lung 60 days post-BMT and found that ICOS^−/−^ donor cells induced less sclerodermatous-like pathology, reflected by retention of subcutaneous fat in the skin and less fibrosis in both skin and lung tissues (Figures 1D,E). These results indicate that ICOS expression on donor grafts promoted the progression of cGVHD.

### ICOS Promotes Treg and Tfh Development in cGVHD

**Figure 2:**
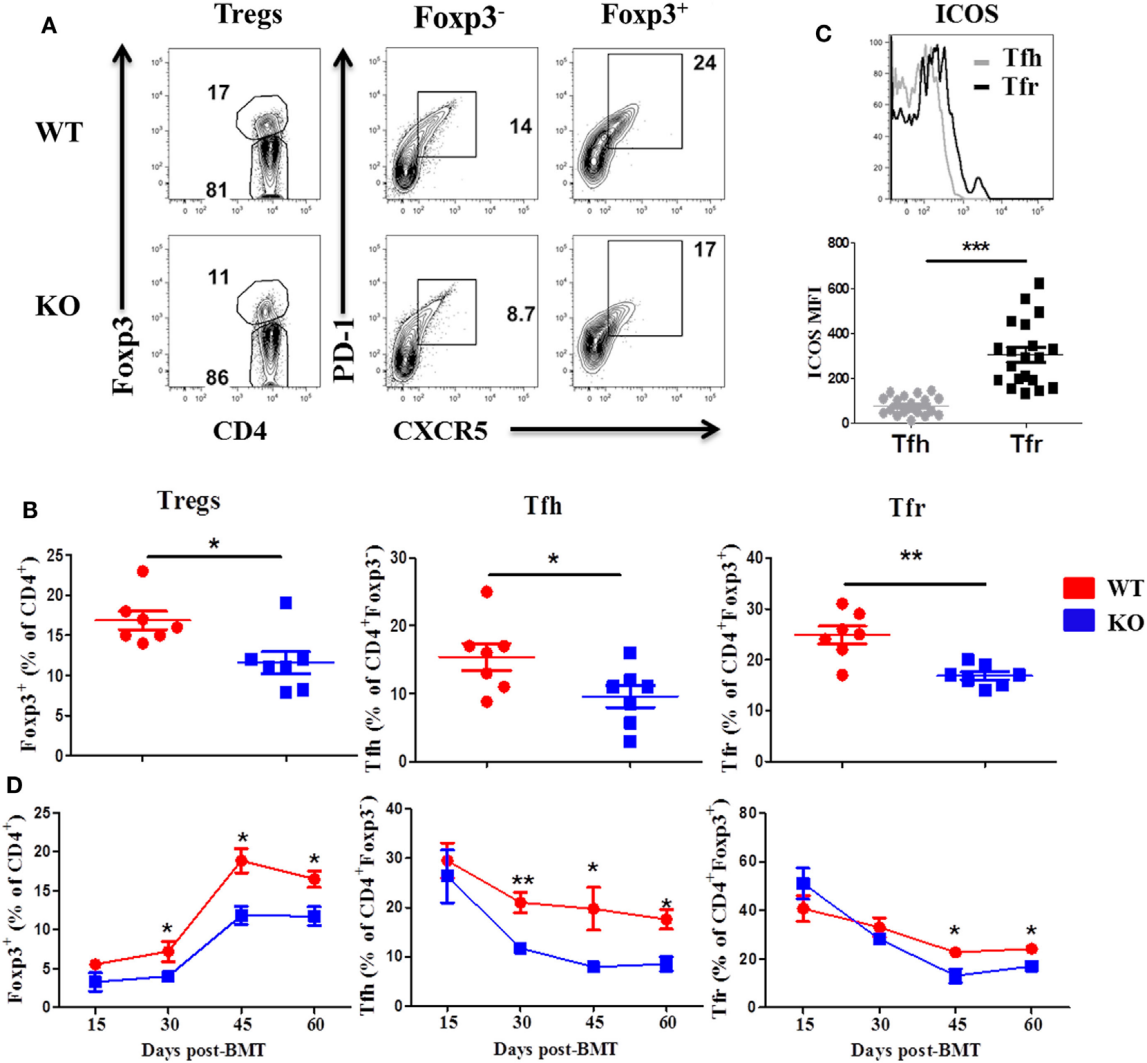
Inducible T-cell co-stimulator (ICOS) promotes development of regulatory T cell (Treg) and T follicular helper (Tfh) cells. Bone marrow transplantation (BMT) was performed as described in Figures 1B–E. 60 days after BMT, recipient spleens were harvested and processed for flow cytometry. Representative contour plots from individual mice **(A)** and dot plots of mean percentage **(B)** of Tregs (Foxp3^+^) on gated donor H-2K^b+^CD4 T cells, Tfh cells (PD-1^+^CXCR5^+^) on gated H-2K^b+^CD4^+^Foxp3^−^, and follicular regulatory T (Tfr) cells (PD-1^+^CXCR5^+^) on gated H-2K^b+^CD4^+^Foxp3^+^ T cells are shown, *n* = 7 mice/group. Representative histogram plots of individual mice from wild-type (WT) group [**(C)** top] and mean fluorescence intensity (MFI) of ICOS on Tfh and Tfr cells are shown [**(C)**, bottom]. BMT parameters were the same as described in Figures 1B–E, at day 15, 30, 45, and 60 post-BMT, spleens were collected and analyzed by flow cytometry. Summary of percentage of Tregs, Tfh, and Tfr are shown over time **(D)**, *n* = 3–4 mice/group. *p < 0.05, **p < 0.01, and ***p < 0.001.

We then examined Treg and Tfh differentiation 60 days post-BMT, as they play critical roles in the development of cGVHD (7). We observed that recipients of ICOS^−/−^ donor grafts had significantly reduced Treg frequency compared with WT controls in the spleen, but not the thymus (Figures 2A,B, and data not shown), suggesting that ICOS influences the generation of iTregs but not the development of nTregs. The recipients of ICOS^−/−^ grafts also had significantly reduced Tfh among CD4^+^Foxp3^−^ and Tfr among CD4^+^Foxp3^+^ cells compared with those of WT grafts (Figures 2A,B). A marked elevation in ICOS expression on Tfr cells was observed compared with that on Tfh cells among WT donor T cells (Figure 2C), suggesting that ICOS may play a greater role in Tfr differentiation than in Tfh cells. Notably, we observed that follicular-like CD8 T cells, which resemble Tfh cells and expressed ICOS, were decreased in the recipients of ICOS^−/−^ donor grafts compared with those of WT grafts (Figure S1A in Supplementary Material), suggesting that ICOS played a role in follicular-like CD8 T cell development during cGVHD pathogenesis.

To further understand how ICOS expression impacts cGVHD, we evaluated the kinetics of Treg, Tfr, and Tfh differentiation in the spleen of recipients at day 15, 30, 45, and 60 after BMT. In the recipients of WT or ICOS^−/−^ donor grafts, Tregs began to gradually increase after day 15, peaked on day 45, and stabilized through day 60 post-BMT. However, Tregs were significantly reduced from day 30 to day 60 in the recipients of ICOS^−/−^ grafts when compared with those of WT (Figure 2D), indicating that ICOS promoted Treg development during cGVHD development. In both groups, Tfh cells among CD4^+^Foxp3^−^ peaked at day 15 and slowly declined through day 60. However, Tfh cells were significantly lower from day 30 to day 60 in the recipients of ICOS^−/−^ grafts (Figure 2D), indicating that ICOS similarly affected Tfh cells development. Tfr cells among CD4^+^Foxp3^+^ retained relatively high levels at day 15, yet gradually decreased over time, and finally sustained a steady state from day 45 to day 60; albeit, significantly fewer Tfr cells were generated from ICOS^−/−^ T cells compared with WT T cells in later stages of cGVHD development (Figure 2D). Taken together, these data indicate that ICOS affects Treg and Tfh differentiation by day 30 after BMT, correlated with disease onset.

### ICOS Promotes Th17 Differentiation

**Figure 3:**
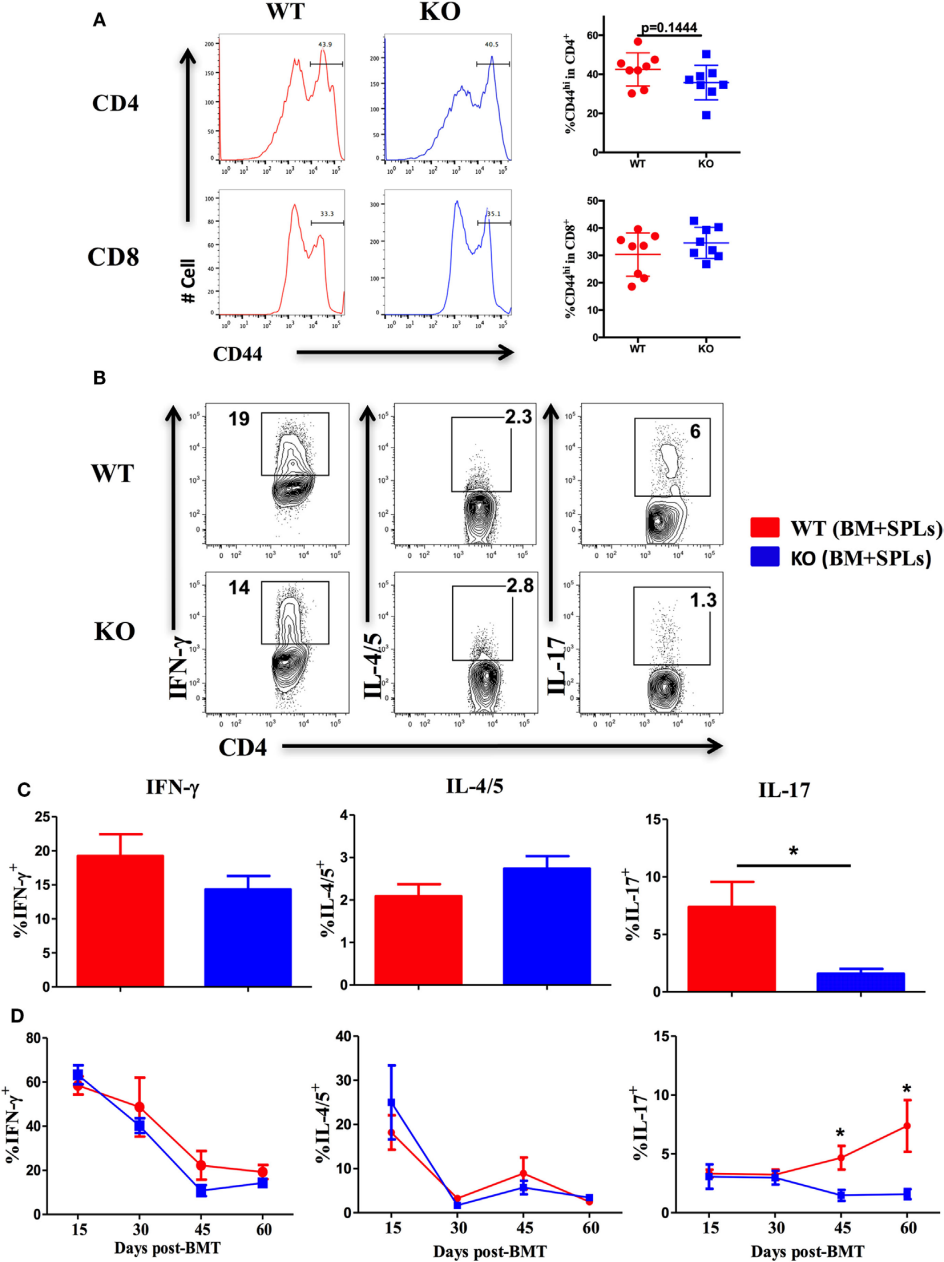
Inducible T-cell co-stimulator promotes Th17 cell differentiation. Bone marrow transplantation (BMT) was setup as described in Figures 1B–E. 60 days after BMT, recipient spleens were harvested and processed for flow cytometry. **(A)** Representative histograms plots and mean percentage of CD44 gated on H-2K^b+^CD4 and CD8 T cells are shown, *n* = 8 mice/group. Representative contour plots of individual mice **(B)** and bar graphs of mean percentage **(C)** of IFN-γ^+^, IL-4/5^+^, and IL-17^+^ on gated H-2K^b+^CD4^+^ cells are shown, *n* = 6–8 mice/group. Summary of mean percentage of IFN-γ^+^, IL-4/5^+^, and IL-17^+^ on gated H-2K^b+^CD4^+^ cells are shown over time **(D)**, *n* = 3–4 mice per group. *p < 0.05.

Given that ICOS controls the memory T-cell pool (25) and is essential for CD4 T cell activation (37), we measured CD44 and CD69 expression on T cells derived from WT and ICOS KO donor grafts. We observed comparable percentages of CD44^+^ cells among CD4 and CD8 T cells (Figure 3A), but a lower percentage of CD69 on CD4 (data not shown), suggesting that ICOS is required for allogeneic T cell activation in cGVHD development.

Effector CD4 T cells drive the pathogenesis of cGVHD (7). ICOS has a distinct role in Th1 and Th2 differentiation depending on disease context (18), but is known to consistently promote Th17 development (19, 41). We therefore tested the impact of ICOS expression on CD4 T-cell differentiation in cGVHD. Upon comparison of cytokine secretion by T cells, we observed similar IFN-γ and IL-4/5 secretion by WT or ICOS^−/−^ CD4 T cells, yet IL-17A production was dramatically decreased by ICOS^−/−^ CD4 T cells (Figures 3B,C). We then assessed kinetics of Th1, Th2, and Th17 cells during cGVHD pathogenesis and found that IFN-γ and IL-4/5 production were again comparable in WT and ICOS^−/−^ T cells (Figure 3D). However, ICOS^−/−^ donor T cells produced lower levels of IL-17A from 45 to 60 days post-BMT when compared with WT T cells (Figure 3D). These results suggested that ICOS was necessary for Th17, but dispensable for Th1 or Th2, differentiation during cGVHD development.

Given that ICOS also promotes CD8 T-cell activation and expansion (42) that can contribute to cGVHD (43), we quantified the effect of ICOS expression on CD8 T cells after BMT. We observed reduced CD69 expression and IFN-γ secretion on donor ICOS^−/−^ CD8 T cells, but not IL-17 (data not shown), which was correlated with reduced cGVHD severity (11). Taken together, ICOS controls both CD4 and CD8 T-cell activation and differentiation in cGVHD pathogenesis.

### ICOS Induces GC B-Cell Development and Plasma Cell Differentiation

Inducible T-cell co-stimulator is required for the differentiation of Tfh cells (23), which promote GC B-cell formation, plasma cell differentiation, and antibody production (18). These activated donor B cells then act as antigen-presenting cells (APCs) to stimulate T cells (44). We next examined the effect of ICOS on donor B-cell activation and differentiation. The recipients of ICOS^−/−^ donor grafts had a lower frequency of Fas^+^GL-7^+^ GC B cells and B220^low^CD138^+^ plasma cells when compared with those of WT grafts (Figure S1B in Supplementary Material). Albeit, B-cell reconstitution and expression of co-stimulatory molecules were comparable (Figure S1B in Supplementary Material and data not shown). These data suggest that ICOS expression on donor T cells promotes GC formation and plasma cell differentiation. In kinetic experiments, we observed fewer GC and plasma cells in the recipients of ICOS^−/−^ donor grafts starting at day 30 post-BMT (data not shown), which correlated with a reduction in Tfh cells during cGVHD development (Figure 2D). We tested serum autoantibody specific for dsDNA and we found significantly lower levels of total IgG, IgG1, and IgG2c in the sera taken from recipients of ICOS^−/−^ grafts (Figure S1C in Supplementary Material). Taken together, ICOS on donor T cells affects B-cell and plasma cell differentiation and antibody production.

### ICOS Promotes Treg Survival and Homeostasis *In Vivo*

**Figure 4:**
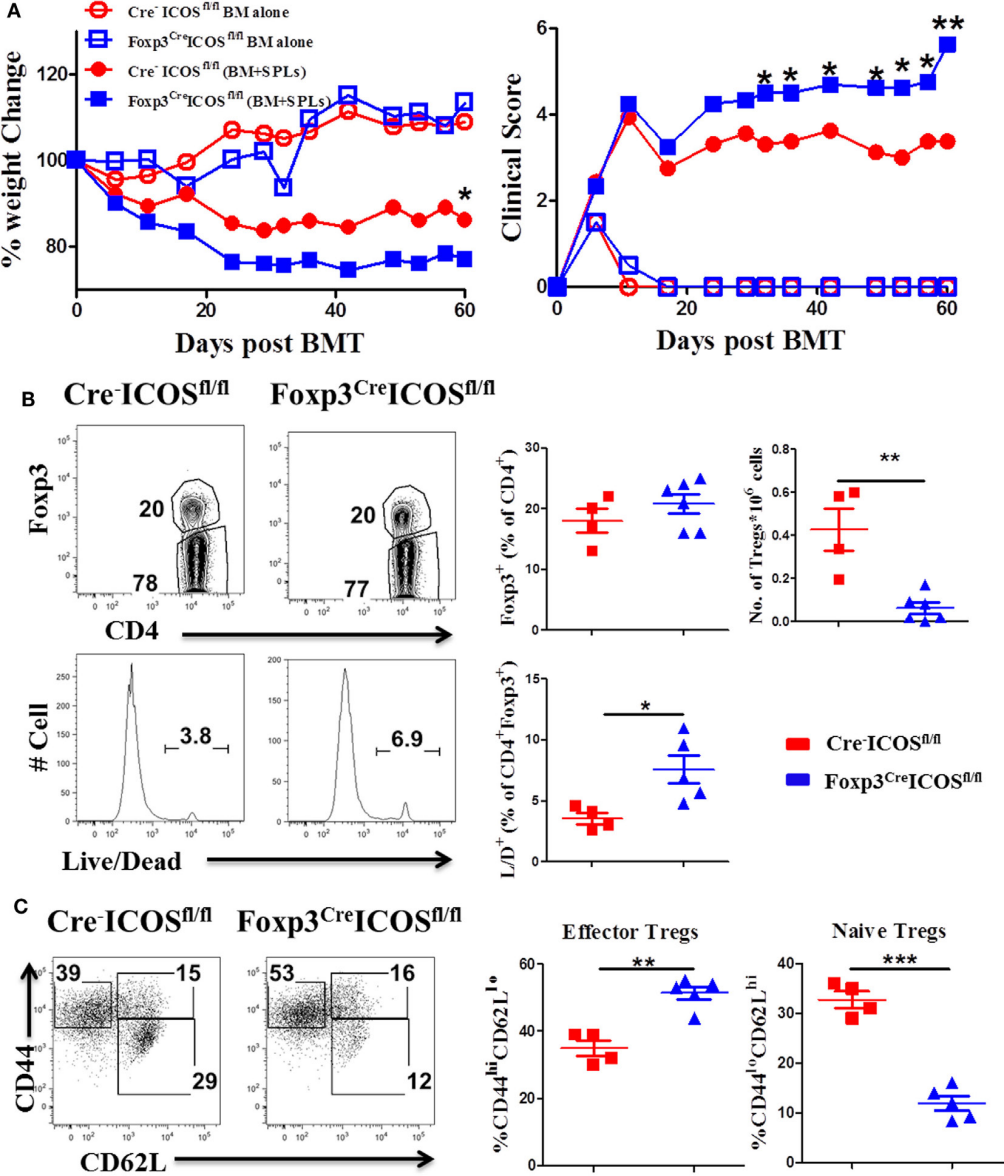
Inducible T-cell co-stimulator (ICOS) is indispensable for regulatory T cells (Tregs) survival and homeostasis in chronic graft-versus-host disease. Lethally irradiated BALB/c mice were transplanted with 0.25 × 10^6^ CD25^−^SPLs and 5 × 10^6^ T-cell-depleted bone marrow from Cre^−^ICOS^fl/fl^ or Foxp3^Cre^ICOS^fl/fl^ mice on B6 background. Body weight and clinical score **(A)** were monitored weekly, *n* = 6–8 mice/group. Spleens were harvested at day 60 after bone marrow transplantation (BMT) and subjected to FACS staining. Representative flow images from individual mice and mean percentage of Foxp3 gated on H-2K^b+^CD4^+^ T cells and live/dead gated on H-2K^b+^CD4^+^ Foxp3^+^ are shown **(B)**. Representative dot plots from individual mice and dot graphs of mean percentage of CD44^hi^CD62L^lo^ and CD44^lo^CD62L^hi^ gated on H2K^b+^CD4^+^Foxp3^+^ T cells are shown **(C)**. *n* = 4–5 mice/group. *p < 0.05, **p < 0.01, and ***p < 0.001.

To further elucidate the role of ICOS in Tregs, we generated mice with a Foxp3-specific ICOS deletion and performed BMT using Foxp3^YFP-Cre^ICOS^fl/fl^ or Cre^−^ICOS^fl/fl^ mice as donors. Given that Foxp3 gene is located on the X chromosome, this allele can be randomly silenced in female mice; we therefore chose male mice as donors to confirm ICOS deletion. Due to decreased CD25^+^Foxp3^+^ cells in Foxp3^YFP-Cre^ICOS^fl/fl^ mice (data not shown), we used CD25-depleted donor splenocytes (CD25^−^SPL) to induce cGVHD. The recipients of CD25^−^SPL from Foxp3^YFP-Cre^ICOS^fl/fl^ donors had more severe cGVHD than those with Cre^−^ICOS^fl/fl^ donors, evidenced by lower body weight maintenance and higher clinical scores (Figure 4A). These data suggest that ICOS is required for optimal Treg development and/or function during cGVHD development. To determine at what stage ICOS affects Treg development, we evaluated Foxp3 expression in the thymus of recipients. We observed that frequencies of Foxp3^+^ among CD4^+^CD8^−^ cells were comparable among cohorts (data not shown), suggesting that ICOS is dispensable for the development of nTregs in recipient thymus. While frequencies of CD4^+^Foxp3^+^ cells were similar in the spleen (Figure 4B), we observed significant reductions in absolute number and survival of Foxp3^+^ cells in the recipients of Foxp3^YFP-Cre^ICOS^fl/fl^ donor grafts when compared with those of Cre^−^ICOS^fl/fl^ grafts (Figure 4B). These data suggest that ICOS promotes Treg survival but not generation in lymphoid organs. In addition, Tregs derived from Foxp3^YFP-Cre^ICOS^fl/fl^ donor cells exhibited an activated phenotype, with significantly higher frequencies of CD44^hi^CD62L^lo^ (effector) cells and lower CD44^lo^CD62L^hi^ (naïve) cells compared with those derived from Cre^−^ICOS^fl/fl^ donor cells (Figure 4C), suggesting that ICOS is critical for maintaining homeostasis of Tregs.

**Figure 5:**
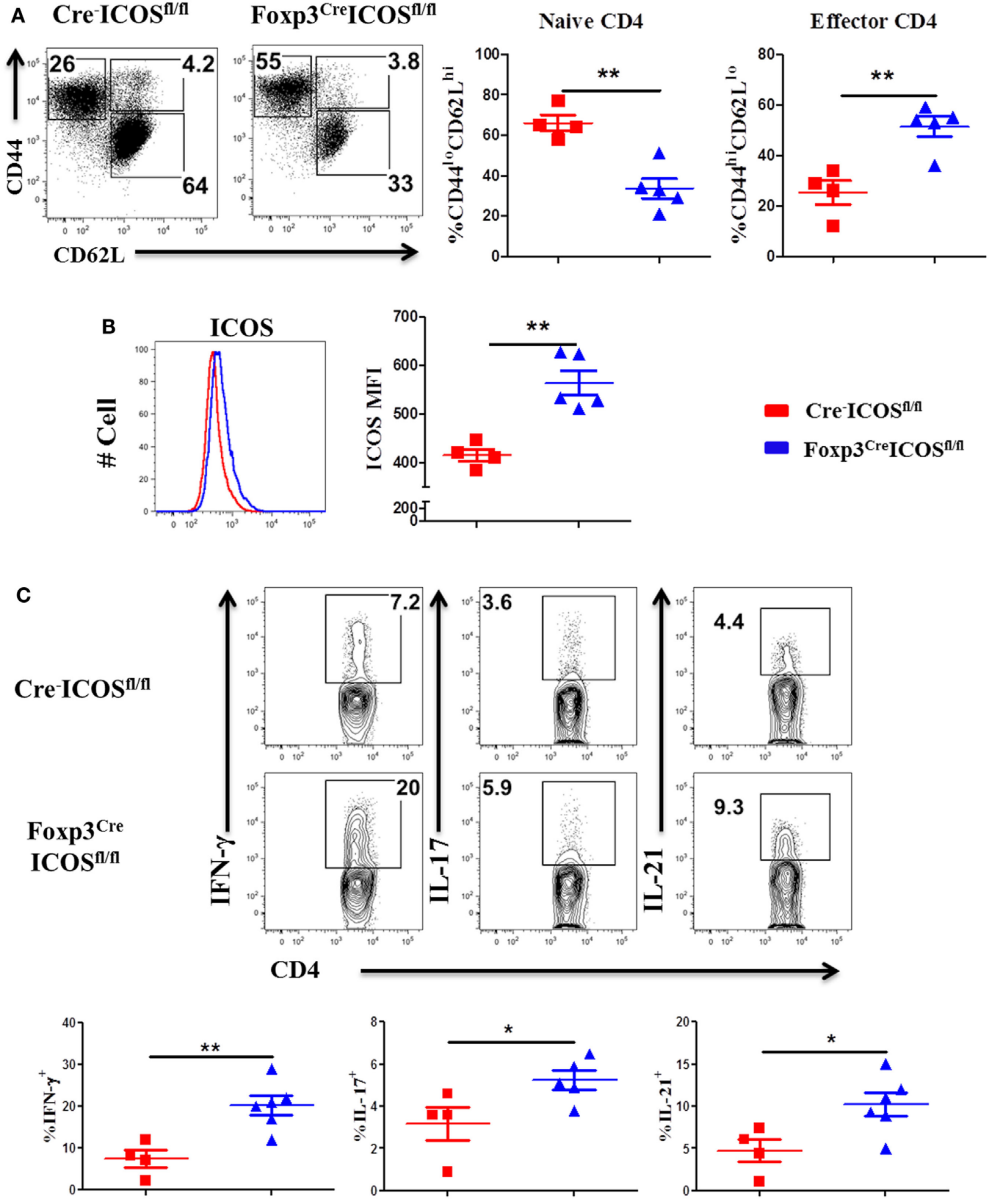
Inducible T-cell co-stimulator (ICOS) on regulatory T cells affects activation of effector T cells. Bone marrow transplantation (BMT) was performed as described in Figure 4. Splenocytes were harvested and processed for flow cytometry 60 days after BMT. Representative dot plots from individual mice and mean percentage of CD44^hi^CD62L^lo^ and CD44^lo^CD62L^hi^ on gated H-2K^b+^CD4^+^ Foxp3^−^ T cells are shown **(A)**. Representative histogram and mean fluorescence intensity (MFI) of ICOS on gated H-2K^b+^CD4^+^Foxp3^−^ T cells are shown **(B)**. *n* = 4–5 mice/group. Single-cell suspension of splenocytes was processed for intracellular cytokine staining. The representative contour plots from individual mice and mean percentage of IFN-γ^+^, IL-4/5^+^, and IL-17^+^ gated on H-2K^b+^CD4^+^ cells are shown **(C)**, *n* = 4–6 mice/group. *p < 0.05 and **p < 0.01.

To corroborate a role of ICOS in effector cell generation, we examined the phenotype of donor T cells and observed that donor CD4 T cells exhibited higher CD44^hi^CD62L^lo^ frequencies (Figure 5A) coupled with higher ICOS expression (Figure 5B) but had lower CD44^lo^CD62L^hi^ frequencies in the recipients of Foxp3^YFP-Cre^ICOS^fl/fl^ (Figure 5A). Moreover, donor CD4 T cells isolated from recipients of Foxp3^YFP-Cre^ICOS^fl/fl^ produced more pro-inflammatory cytokines, including IFN-γ, IL-17, and IL-21 (Figure 5C). Similar results were observed in donor CD8 T cells, although less dramatic (data not shown). These results suggest that ICOS may be required for optimal suppressive function of Tregs.

### ICOS Promotes Tfr Development *In Vivo*

**Figure 6:**
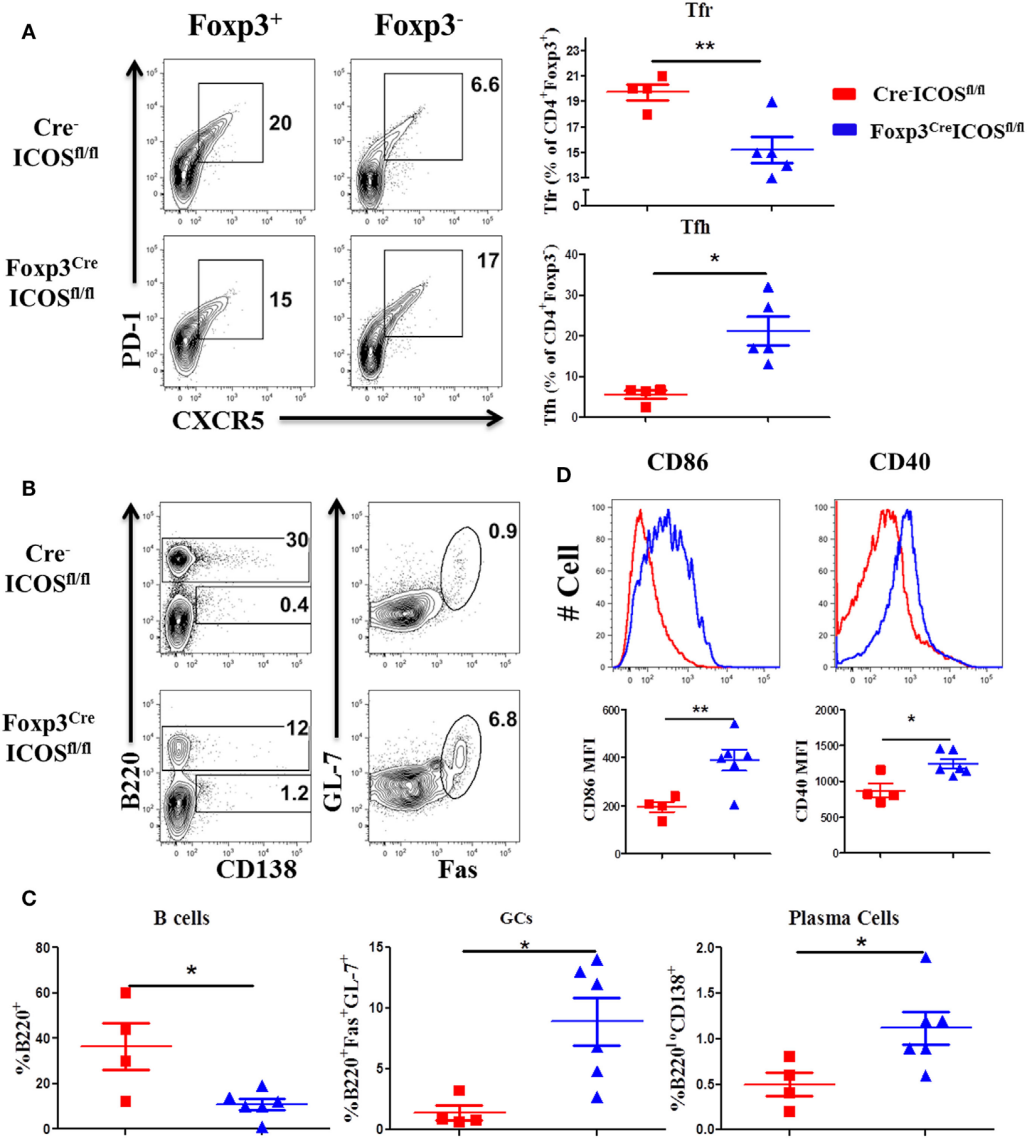
Inducible T-cell co-stimulator (ICOS) promotes follicular regulatory T (Tfr) development. Bone marrow transplantation (BMT) was setup as described in Figure 4. Splenocytes were analyzed by flow cytometry at 60 days after BMT. Representative contour plots of individual mice and mean percentage of T follicular helper (Tfh) cells (PD-1^+^CXCR5^+^) on gated H-2K^b+^CD4^+^Foxp3^−^ and Tfr cells (PD-1^+^CXCR5^+^) on gated H-2K^b+^CD4^+^Foxp3^+^T cells are shown **(A)**, *n* = 4–5 mice/group. Representative contours plots of each group and mean percentage of B220^+^ and B220^−^CD138^+^ plasma cells on gated H-2K^b+^ cells, GL-7^+^Fas^+^ germinal center B cells (GCs) gated on H-2K^b+^B220^+^ cells are shown **(B,C)**, *n* = 4–6 mice/group. Representative histograms and mean fluorescence intensity (MFI) of CD86 and CD40 gated on H-2K^b+^B220^+^ cells are shown **(D)**. *p < 0.05 and **p < 0.01.

Given the requirement for ICOS in Tfr cells (22), we compared the presence of Tfh and Tfr cells in the recipients of Foxp3^YFP-Cre^ICOS^fl/fl^ or Cre^−^ICOS^fl/fl^ donor grafts. We observed a higher percentage of Tfh among CD4^+^Foxp3^−^ cells, but lower percentage of Tfr among CD4^+^Foxp3^+^ cells, in recipients of Foxp3^YFP-Cre^ICOS^fl/fl^ grafts (Figure 6A). Consistently, B-cell reconstitution (B220^+^) was significantly decreased (Figures 6B,C), whereas B-cell activation (CD40 and CD86 expression) (Figure 6D) and GC B and plasma cell differentiation were significantly increased (Figures 6B,C), suggesting that ICOS is required for Tfr cells to inhibit B-cell activation and differentiation. In addition, we observed that the percentages of follicular-like CD8 T cells increased and secreted more IL-21 in ICOS-deficient donor Tregs (Figures S2A,B in Supplementary Material). This suggests that Tfr cells can inhibit follicular-like CD8 T cells that promote B-cell differentiation. Overall, these results indicate that ICOS is indispensable for Tfr development and suppressive function.

### ICOS Is Required for the Optimal Function and Stability of iTregs *In Vivo*

**Figure 7:**
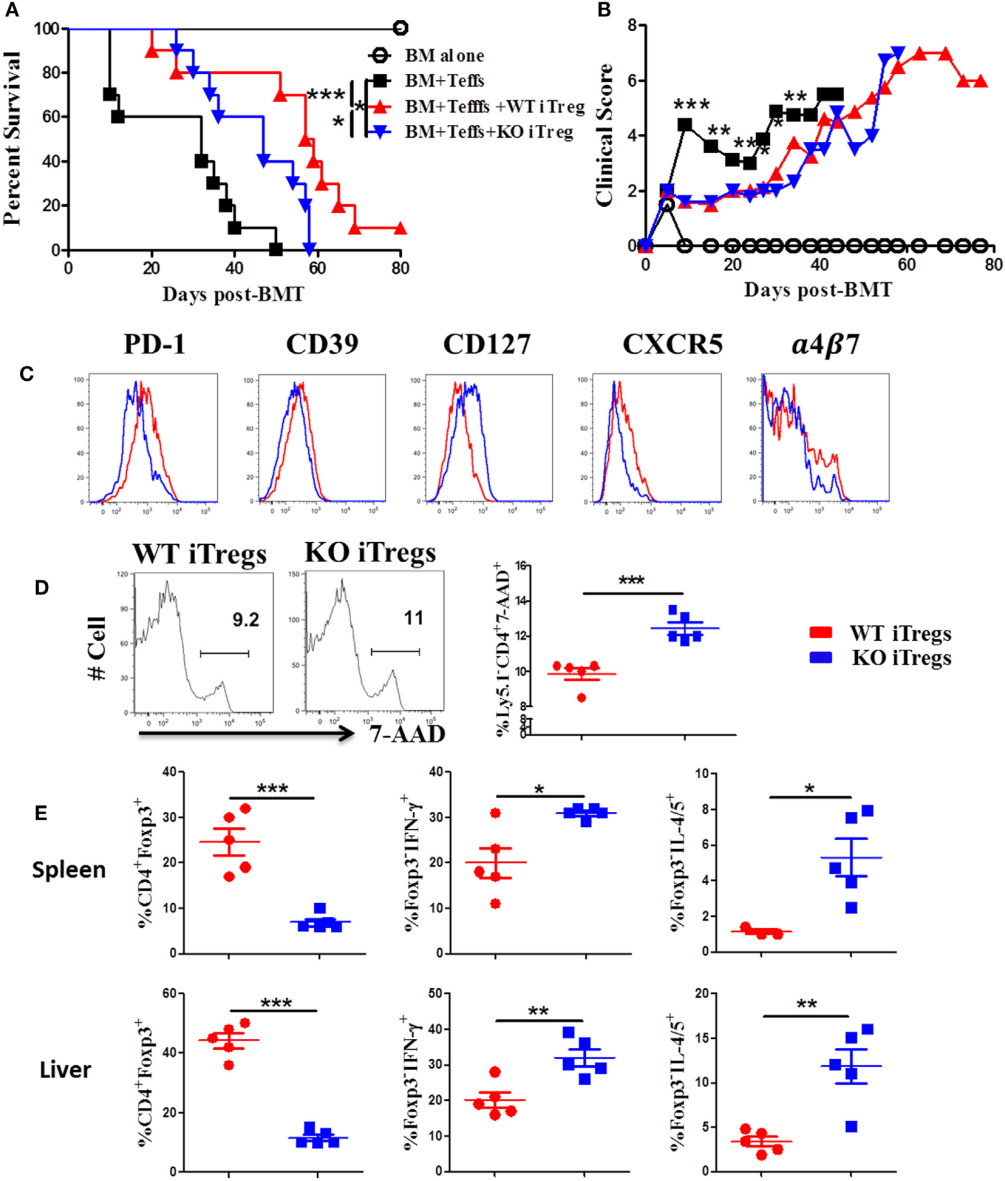
Inducible T-cell co-stimulator (ICOS) is required for optimal function and stability of iTregs *in vivo*. Lethally irradiated BALB/c mice were transplanted with 5 × 10^6^ BM from Rag1^−/−^ B6 plus 0.5 × 10^6^ CD25^hi^CD4^+^ cells isolated from wild-type (WT) or ICOS^−/−^ mice (Ly5.1^−^). Three days later, 0.5 × 10^6^ CD25^−^Ly5.1^+^ B6 Teffs were injected into each recipient. Recipients were monitored for survival **(A)** and body weight loss **(B)** for 80 days, *n* = 10 mice/group. In separate experiments, spleens and livers were excised and processed for FACS staining on day 14 after allo-bone marrow transplantation (BMT). Histograms of PD-1, CD39, CD127, CXCR5, and α4β7 expressed on gated H-2K^b+^Ly5.1^−^CD4^+^Foxp3^+^ in spleen are shown **(C)**. Representative histograms from individual mice and mean percentage of 7-AAD on gated H-2K^b+^Ly5.1^−^CD4^+^Foxp3^+^ are shown **(D)**. Mean percentage of CD4^+^Foxp3^+^ gated on H-2K^b+^Ly5.1^−^, and Foxp3^−^IFN-γ^+^ and Foxp3^−^IL-4/5^+^ gated on H-2K^b+^Ly5.1^−^CD4^+^ cells from spleens and livers are shown **(E)**. *n* = 5 mice/group. *p < 0.05, **p < 0.01, and ***p < 0.001.

To further test how ICOS impacts stability and function of alloantigen-reactive iTregs, we stimulated CD25-depleted CD4 T cells from WT or ICOS KO mice with allogeneic APCs under Treg-polarization conditions as previously described (45). We then compared the suppressive function of iTregs between groups pertaining to their ability to suppress the induction of GVHD when co-transplanted with CD25^−^ WT T cells. While WT iTregs were able to significantly alleviate GVHD, ICOS KO iTregs were compromised (Figures 7A,B). These results suggest that ICOS is required for the optimal function of iTregs to suppress GVHD development.

To understand the underlying mechanism, we measured molecular markers related to Treg function. When compared with WT counterparts, ICOS^−/−^ iTregs had lower expression of PD-1 and CD39, both known to be positively correlated with Treg suppressive function (46, 47), and a higher expression of CD127, conversely known to negatively impact Treg suppressive function (48) and stability (49) (Figure 7C). However, ICOS had a little effect on the expression of CD73, CTLA-4, GITR, and Nrp1 (data not shown). Chemokine receptors are important for Treg migration into areas of inflammation (50). We observed that ICOS^−/−^ iTregs expressed lower levels of chemokine receptor CXCR5 and gut-homing adhesion molecule α4β7 (Figure 7C), but did not affect expression of CCR4, CCR5, or CCR9 (data not shown). These data suggest that ICOS promotes iTreg migration to target organs. ICOS^−/−^ iTregs also displayed higher frequencies of 7-AAD^+^ cells compared with WT iTregs in recipient spleens (Figure 7D), suggesting that ICOS is crucial for the survival of iTregs *in vivo*. Furthermore, ICOS^−/−^ iTregs exhibited significantly lower percentages of Foxp3, yet higher IFN-γ or IL-4/5 in recipient spleen and liver (Figure 7E), suggesting that iTregs are more prone to lose Foxp3 and subsequently differentiate into Th1 or 2 cells.

We then directly compared the suppressive capacity of WT or ICOS^−/−^ iTregs on day 14 post-BMT. We found that WT iTregs had a greater capacity to suppress donor CD4 Teffs (Ly5.1^+^) (Figure S3A in Supplementary Material), but not Ly5.1^+^CD8^+^ T cells (data not shown). In addition, WT iTregs induced significantly more death on Teffs than ICOS^−/−^ iTregs (Figure S3B in Supplementary Material), suggesting that ICOS facilitates iTreg-mediated death of Teffs. We further evaluated the pro-inflammatory cytokines produced by Teffs and found that ICOS was required for iTregs to inhibit IFN-γ, but not IL-17, production by donor CD4 Teffs (Figure S3C in Supplementary Material). Taken together, these results suggest that ICOS is required for the survival, stability, function, and migration of Tregs *in vivo*.

### Treatment of Anti-ICOS Antibody Alleviates cGVHD

**Figure 8:**
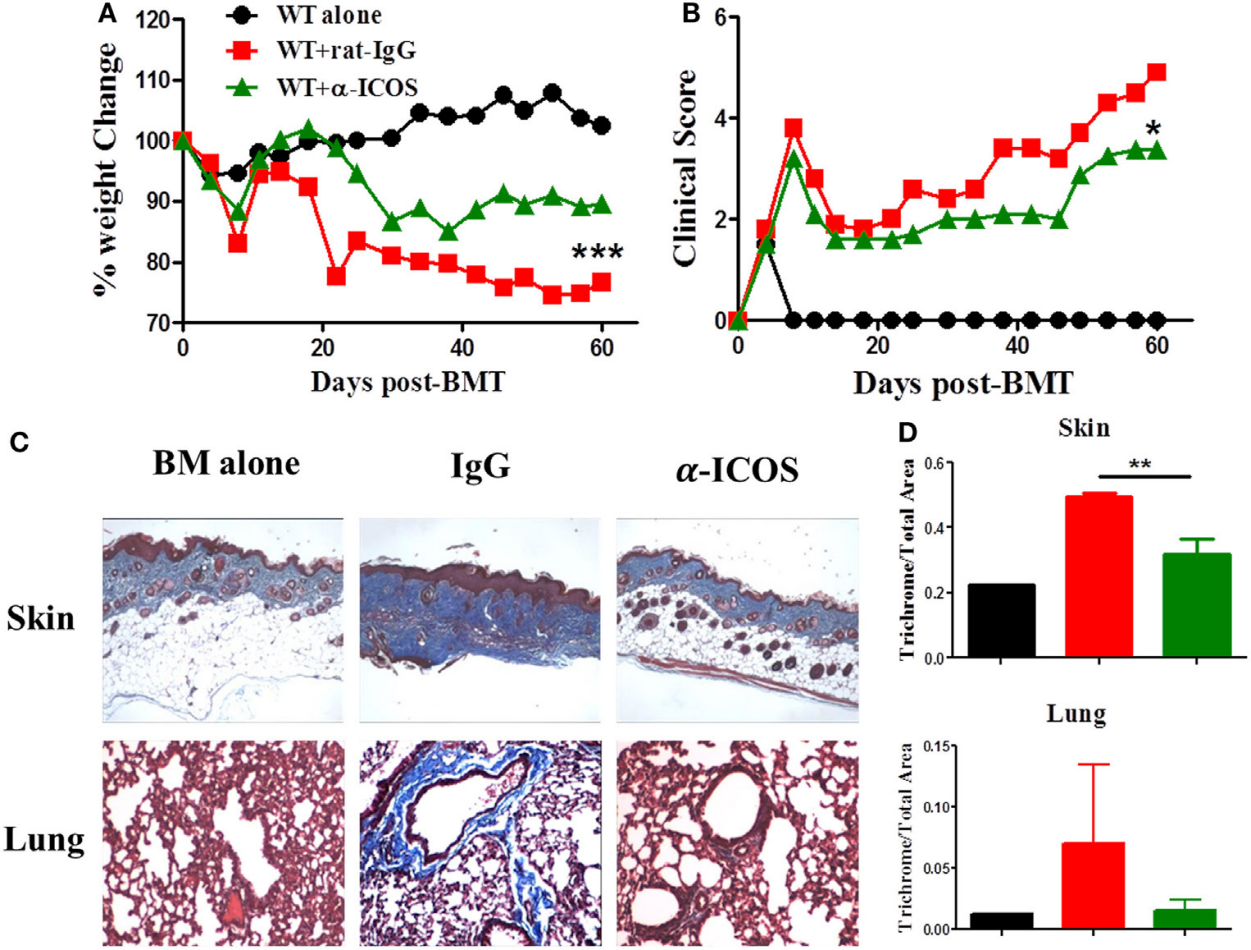
Treatment with anti-inducible T-cell co-stimulator (ICOS) antibodies alleviates chronic graft-versus-host disease. Lethally irradiated BALB/c mice were transferred with 5 × 10^6^ T-cell-depleted bone marrow and 0.5 × 10^6^ SPLs on B6 background. Anti-ICOS Abs were administrated at 200 μg/mouse 3 times/week from day 0 to day 28 after bone marrow transplantation (BMT). Body weight maintenance **(A)** and clinical score **(B)** were monitored for 60 days, *n* = 5 mice/group. Representative pictures of skin and lung stained for collagen 60 days after transplantation are shown **(C)**. Bar graphs of ratios of collagen area to total areas of skin and lung qualified by ImageJ are shown **(D)**. *p < 0.05, **p < 0.01, and ***p < 0.001.

**Figure 9:**
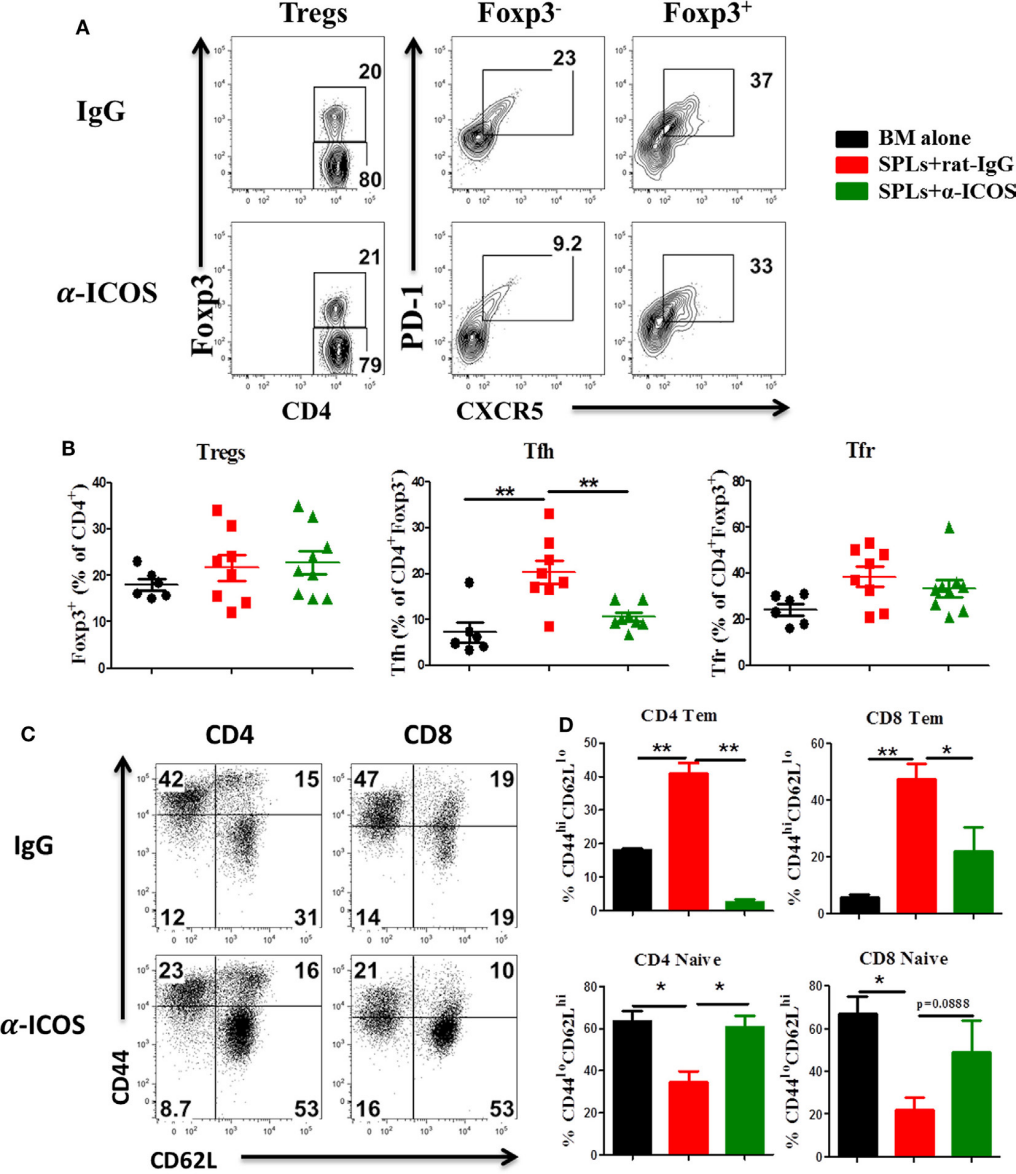
Anti-inducible T-cell co-stimulator (ICOS) antibody treatment reduces effector T cells differentiation. Bone marrow transplantation (BMT) was performed as described in Figure 8. Splenocytes were stained and analyzed by flow cytometry at day 60 after BMT. Representative contour plots **(A)** and mean percentage **(B)** of regulatory T cells (Tregs) (CD4^+^Foxp3^+^) on gated donor H-2K^b+^ T cells, T follicular helper (Tfh) cells (PD-1^+^CXCR5^+^) on gated H-2K^b+^CD4^+^Foxp3^−^, and follicular regulatory T (Tfr) cells (PD-1^+^CXCR5^+^) on gated H-2K^b+^CD4^+^Foxp3^+^ T cells are shown, two experiments were pooled together, *n* = 8–9 mice/group. Representative dot plots **(C)** and mean percentage **(D)** of CD44^hi^CD62L^lo^ and CD44^lo^CD62L^hi^ on gated H-2K^b+^CD4^+^ and H-2K^b+^CD8^+^ T cells are shown, *n* = 3–4 mice/group. *p < 0.05 and **p < 0.01.

To determine the feasibility of clinical translation, we next evaluated whether pharmacologically blocking ICOS could attenuate cGVHD severity. As Treg development peaked at day 45 after BMT (Figure 2D) and ICOS was required for Treg fitness (Figure 4), we chose to administer α-ICOS mAb from day 0 to day 28. When compared with rat-IgG, α-ICOS treatment significantly reduced cGVHD severity, reflected by better body weight maintenance and lower clinical scores (Figures 8A,B). Accordingly, α-ICOS treatment reduced fibrosis in recipient skin and lung (Figures 8C,D). To investigate the underlying mechanisms, we evaluated the effects of anti-ICOS on donor T-cell activation and differentiation on day 60 post-BMT. Indeed, treatment with α-ICOS reduced Tfh differentiation, but had no effect on Treg and Tfr (Figures 9A,B). This was consistent with preserved thymic function as reflected by percentages of CD4^+^CD8^+^ cells (Figure S4A in Supplementary Material). The recipients treated with α-ICOS also had decreased memory T (CD44^hi^CD62L^lo^) and increased naïve T-cell (CD44^lo^CD62L^hi^) frequencies (Figures 9C,D), which are known to be negatively and positively related with cGVHD severity, respectively (43, 51). Furthermore, we observed that α-ICOS treatment improved B-cell reconstitution as reflected by increased frequencies of donor B220^+^ cells (Figures S4B,C in Supplementary Material), and reduced B-cell activation reflected by lower expression of CD86 (Figure S4D in Supplementary Material). However, α-ICOS treatment did not affect B-cell differentiation into GC and plasma cells. Taken together, α-ICOS treatment after BMT improved cGVHD outcomes by decreasing Teff-cell differentiation while restoring normal B-cell homeostasis and, importantly, by preserving thymic function and Treg development.

## Discussion

In this study, we demonstrate a critical role for ICOS in Teffs and Tregs, as well as B cells, in the pathogenesis of cGVHD. ICOS promoted cGVHD by boosting pathogenic T cells, pro-inflammatory cytokine production, and B-cell differentiation. Conversely, ICOS also facilitated Treg and Tfr development to restrict aberrant T and B cell responses and thus alleviate cGVHD. ICOS was required for Treg development, survival, and homeostasis in cGVHD development. Furthermore, ICOS blockade attenuated the severity of cGVHD by impeding the T-cell response and consequently B-cell differentiation; yet not affecting Treg development. In addition, we implicate follicular-like CD8 T cells, which are inhibited by Tfr, as a contributor to the pathogenic T cell pool in mediating cGVHD, and demonstrated that these cells required ICOS for differentiation. In summary, we found ICOS played a vital role in mediating cGVHD by regulating T and B cell differentiation and response, and that inhibiting ICOS could decrease cGVHD severity and spare Treg development.

It has been reported that ICOS promotes IFN-γ but reduce IL-4 production during the development of aGVHD (30). However, ICOS did not play a significant role in Th1 and Th2 differentiation during cGVHD development. These different results were likely due to the distinct pathophysiology of acute versus chronic GVHD (7). We observed that ICOS^−/−^ donor T cells produced significantly lower IL-17 at day 45 but not in earlier stages (Figure 3), which was consistent with reports showing that ICOS is necessary for Th17 development and maintenance (41). Several mechanisms may account for this: (i) ICOS sustained IL-23R expression on Th17 cells through c-Maf (41, 52), (ii) IL-17 secretion was maintained through ICOS-mediated activation of PI3K pathway (21), and (iii) ICOS promoted IL-21 production by Th17 and Tfh cells (41) that maintain Stat3 activation to sustain the Th17 lineage (53, 54). ICOS^−/−^ T cells also have reduced frequencies of Tfh cells and are unable to upregulate B-cell lymphoma 6 (Bcl-6) and c-Maf expression (22, 23). Consistently, we observed that ICOS controlled Tfh cell differentiation during cGVHD development (Figure 2). In kinetic experiments, ICOS had a dominant effect on Tfh differentiation 30 days after BMT. We interpret that CD28 regulated early Tfh differentiation, whereas ICOS maintained Tfh phenotype and homing to follicle areas by downregulating Kruppel-like factor2 (55).

Inducible T-cell co-stimulator is required for CD8 T-cell activation and IFN-γ secretion, in part because ICOS triggers IL-2 production (37, 42). Surprisingly, we observed that follicular-like CD8 T cells may also contribute to cGVHD pathogenesis. This population has been reported in models of chronic LCMV infection, which demonstrate secretion of pro-inflammatory cytokines and co-expression of CD28 and ICOS (56). Similarly, we observed that ICOS affects follicular-like CD8 T differentiation (Figure S1A in Supplementary Material). Quigley et al. reported that follicular CD8 T cells were localized in tonsil B cell follicles and supported B cell survival (57). Our data suggest that follicular-like CD8 T cells may function in a way akin to Tfh, specifically through IL-21 (Figure S2 in Supplementary Material)-mediated B-cell differentiation. Nonetheless, more studies are required to confirm the contribution of follicular-like CD8 T cells in cGVHD pathogenesis.

Aberrant donor B-cell differentiation is responsible for cGVHD development (58). Similar impairments in B-cell responses have been reported in both ICOS-deficient mice and humans (27, 28). We also found that the recipients of ICOS^−/−^ donor grafts showed decreased GC development, plasma cell differentiation, and Ig production, which correlated with reduced Tfh cells during cGVHD development. These results confirm that ICOS is necessary to drive Tfh function and subsequently support B-cell differentiation likely *via* cell–cell contact (ICOS:ICOSL) (59) and IL-21 secretion (41, 60–62). ICOS was also shown to regulate extrafollicular Tfh cells that can induce B-cell differentiation into short-lived plasma cells, which then produce autoantibodies in a murine lupus model (63). Thus, we cannot exclude the possibility that a defect in extrafollicular Tfh cell function in the absence of ICOS was responsible for the observed impairment in B-cell response.

Previous studies indicate that ICOS controls Treg development and homeostasis (25). Consistently, we observed lower percentages of Tregs upon transplant with ICOS^−/−^ donor T cells (Figure 2). Furthermore, ICOS was not only required for Treg generation but also for Treg homeostasis and survival (Figure 4), which could because ICOS promotes Treg sensitivity to IL-2 and hence resulting in better survival and Foxp3 stability (64, 65). ICOS^−/−^ Tregs could not control the increased activation and differentiation of Teffs (Figure 5), indicating that ICOS was important for Treg suppressive function, possibly though impaired secretion of the suppressive cytokine IL-10 (24, 66).

Consistent with the improvement in aGVHD observed using anti-ICOS treatment (31), prophylactic anti-ICOS treatment also reduced cGVHD severity by decreasing effector T cells without affecting Treg development (Figures 8 and 9). Given ICOS had an impact on Treg generation within 30 days post-BMT, we interpret that prophylactic treatment still allowed Treg generation in the later stages of cGVHD development. Flynn et al. reported that anti-ICOS treatment was sufficient to reverse established cGVHD when administered from day 28 to day 56 post-BMT in a B6 to B10.BR murine model (32). Although the mechanisms accounting for this discrepancy are not clear, we postulate that different BMT models likely contributed to this, rather a *de novo* bronchiolitis cGVHD versus aGVHD to cGVHD transition model.

**Figure 10:**
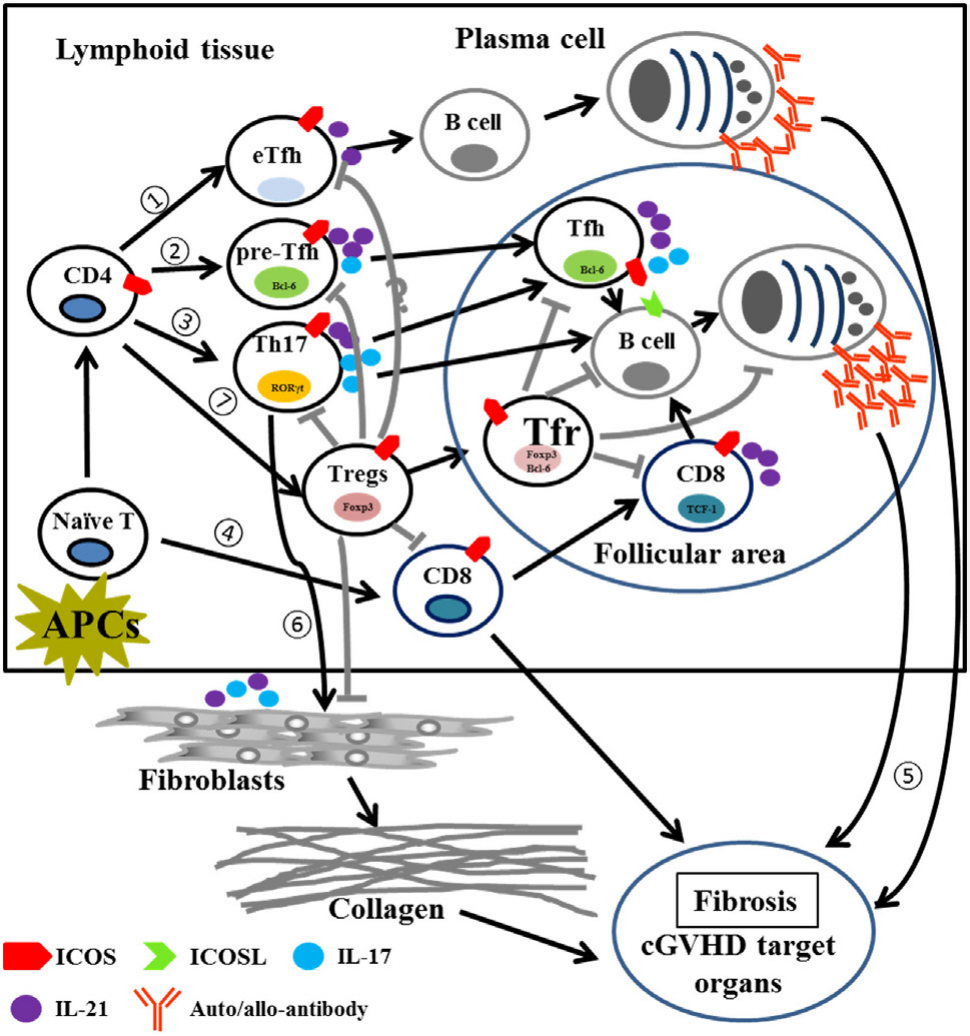
Proposed model for the role of ICOS in pathogenic T cells and Tregs in cGVHD. Abbreviations: APCs, antigen-presenting cells; eTfh, extrafollicular T helper cells; Bcl-6, B-cell lymphoma 6; Foxp3, forkhead box P3; RORγt, retinoic acid receptor-related (RAR) orphan; TCF-1, T cell factor 1; cGVHD, chronic graft-versus-host disease; ICOS, inducible T-cell co-stimulator; Tregs, regulatory T cells.

This study demonstrates that ICOS plays pleiotropic roles in the pathogenesis of cGVHD (Table S1 in Supplementary Material). Post allo-HCT, donor T cells activated *via* interaction with host and/or donor APCs upregulate ICOS in lymphoid tissues. (1) Activated CD4 T cells begin to differentiate into extrafollicular Tfh cells and promote B-cell maturation to short-lived plasma cells that produce auto- or allo-antibodies in the extrafollicular areas. (2) ICOS signaling promotes activated CD4 T cells to express CXCR5 and Bcl-6 and become pre-Tfh cells, which migrate into the follicular area and support B-cell differentiation into long-lived plasma cells by secreting IL-21 and IL-17. (3) Activated T cells also induce Th17 differentiation which produce IL-17 and IL-21 to promote Tfh function and B-cell differentiation into long-lived plasma cells. (4) In response to ICOS signaling, CD8 T cells begin to differentiate and expand; activated CD8 T cells secrete cytokines and/or cytotoxic molecules to induce cGVHD and express CXCR5 and PD-1 which then migrate to follicular areas to promote B cell differentiation into long-lived plasma cells. (5) These short-lived and/or long-lived plasma cells produce auto- and allo-antibodies that deposit into cGVHD target organs to induce cGVHD. (6) Th17 cells secrete cytokines such as IL-17 and IL-21 to facilitate fibroblast maturation and collagen production that subsequently deposits in target organs during cGVHD. (7) On the other hand, ICOS also promotes CD4 T cell differentiation into Tregs, which suppress pre-Tfh, Th17, and CD8 T cells, as well as fibroblasts through cell–cell contact (ICOS:ICOSL) supplemented by inhibitory cytokine secretion (TGF-β and IL-10); ICOS can also induce CXCR5 and Bcl-6 expression on Tregs and promote Tregs to migrate into follicular areas, dubbed Tfr cells, which inhibit Tfh, B cells responses, as well as plasma cells and antibody production (Figure 10). Although Treg and Tfr cells suppress pathogenic T and B cells, they cannot completely contain this response, thus the resultant effect of ICOS expression is exacerbated cGVHD. Our data provide rational to target ICOS for cGVHD prophylaxis in clinic, despite its pluralistic role in T-cell activation and differentiation.

## Ethics Statement

This study was carried out in accordance with the recommendations of “NIH Guide for Care and Use of Laboratory Animal, Institutional Animal Care and Use of Committee.” The protocol was approved by the “Institutional Animal Care and Use of Committee.”

## Author Contributions

MZ participated in experimental design, performed research, collected, analyzed and interpreted data, performed statistical analysis, and drafted and revised the manuscript. YW, DB, SI, JC, AD, HN, MD, SS, and MS performed research, collected and analyzed data, and edited the manuscript. FC interpreted data and edited manuscript. W-KS participated in experiment design, interpreted data, and edited the manuscript. X-ZY designed research, interpreted data, and revised the manuscript. All the authors read and approved the submitted version.

## Conflict of Interest Statement

The authors declare that the research was conducted in the absence of any commercial or financial relationships that could be construed as a potential conflict of interest.
