# Supplementary material for: Inducible T-Cell Co-Stimulator Impacts Chronic Graft-Versus-Host Disease by Regulating Both Pathogenic and Regulatory T Cells
Source: Front Immunol. 2018 Jun 22;9:1461. doi: 10.3389/fimmu.2018.01461 (PMC6023972; doi:10.3389/fimmu.2018.01461)
Supplement: Supplementary file 1 [file data_sheet_1.PDF]

Supporting Information

Supplemental figure legends

Supplemental Figure 1. ICOS induces follicular-like CD8 T cells and B cell differentiation. BALB/c mice were lethally irradiated and transferred with 0.25×10^6 $CD25^-$SPLs and 5×10^6 TCD-BM from WT or $ICOS^{-/-}$ mice on B6 background. Splenocytes were processed and analyzed by flow cytometry. Representative contour plots and mean percentage of follicular-like CD8 T cells ($PD\text{-}1^+CXCR5^+$) on gated $H\text{-}2K^{b+}$CD8 donor cells are shown (**A**). Representative flow images and mean percentage of $B220^+$ and $B220^-CD138^+$ plasma cells on gated $H\text{-}2K^{b+}$ cells, $GL\text{-}7^+Fas^+$ GCs on gated $H\text{-}2K^{b+}B220^+$cells are shown (**B**). Recipient whole blood was collected and serum was tested for anti-dsDNA IgG, IgG1 and IgG2c autoantibodies using ELISA as described in Material and Method, bar graphs of anti-ds DNA IgG, IgG1, and IgG2c are shown (**C**), n=6-8 mice/group. *p<0.05, **p<0.01.

Supplemental Figure 2. ICOS promotes Tfr to suppress follicular-like CD8 T cell development. Lethally irradiated BALB/c mice were transferred with 0.25×10^6 $CD25^-$SPLs and 5×10^6 TCD-BM from $Cre^-ICOS^{fl/fl}$ or $Foxp3^{Cre}ICOS^{fl/fl}$ mice on B6 background. Splenocytes were harvested and processed for flow cytometry 60 days after BMT. Representative flow images and mean percentage of follicular-like CD8 T cells ($CD8^+PD\text{-}1^+CXCR5^+$) on gated donor $H\text{-}2K^{b+}$ cells, and $IL\text{-}21^+$ gated on $H\text{-}2K^{b+}CD8^+PD\text{-}1^+CXCR5^+$ T cells are shown (**A and B**), n=4-6 mice/group. **p<0.01.

Supplemental Figure 3. The suppressive ability of $ICOS^{-/-}$ iTregs was partially impaired in vivo. Lethally irradiated BALB/c mice were transplanted with 5×10^6 BM from $Rag1^{-/-}$ B6 plus 0.5×10^6 $CD25^{hi}CD4^+$ cells isolated from WT or $ICOS^{-/-}$ mice ($Ly5.1^-$). Three days later, 0.5×10^6 $CD25^-Ly5.1^+$ B6 Teffs were injected into each recipient. Single splenocytes were analyzed by flow cytometry. Representative contour plots and mean percentage of $Ly5.1^+$ on gated donor $H\text{-}2K^{b+}$cells, and $CD4^+$ on gated $H\text{-}2K^{b+}Ly5.1^+$ cells are shown (**A**). Representative histograms and mean percentage of 7-AAD on gated $H\text{-}2K^{b+}Ly5.1^+CD4^+$ and $H\text{-}2K^{b+}Ly5.1^+CD8^+$ T cells are shown (**B**). Representative contour plots and mean percentage of IFN-γ and IL-17 on gated $H\text{-}2K^{b+}Ly5.1^+CD4^+$ T cells are shown (**C**). n=3-4 mice/group. *p<0.05, **p<0.01, ***p<0.001.

Supplemental Figure 4. Treatment with anti-ICOS antibodies reduced thymic damage and B cell reconstitution. Lethally irradiated BALB/c mice were transferred with 5×10^6 TCD-BM and 0.5×10^6 SPLs. Anti-ICOS Abs were administrated at 200μg/mouse 3 times per week from day 0 to day 28 after BMT. Single splenocytes and thymocytes were harvested and processed for flow cytometry. Representative flow images and mean percentage of $CD4^+CD8^+$ on gated $H\text{-}2K^{d-}$ cells are shown (**A**). Representative contour plots and scatter plot graphs showing $B220^+$ and $B220^-CD138^+$ plasma cells on gated $H\text{-}2K^{b+}$ cells, $GL\text{-}7^+Fas^+$ GCs on gated $H\text{-}2K^{b+}B220^+$ cells are shown (**B and C**). Histogram of CD86 on gated $H\text{-}2K^{b+}B220^+$cells is shown (**D**). n=6-9 mice/group. *p<0.05, **p<0.01, ***p<0.001.

Supplemental Table 1: Summary of how ICOS impacts immune cells in cGVHD

Supplemental Figure 1

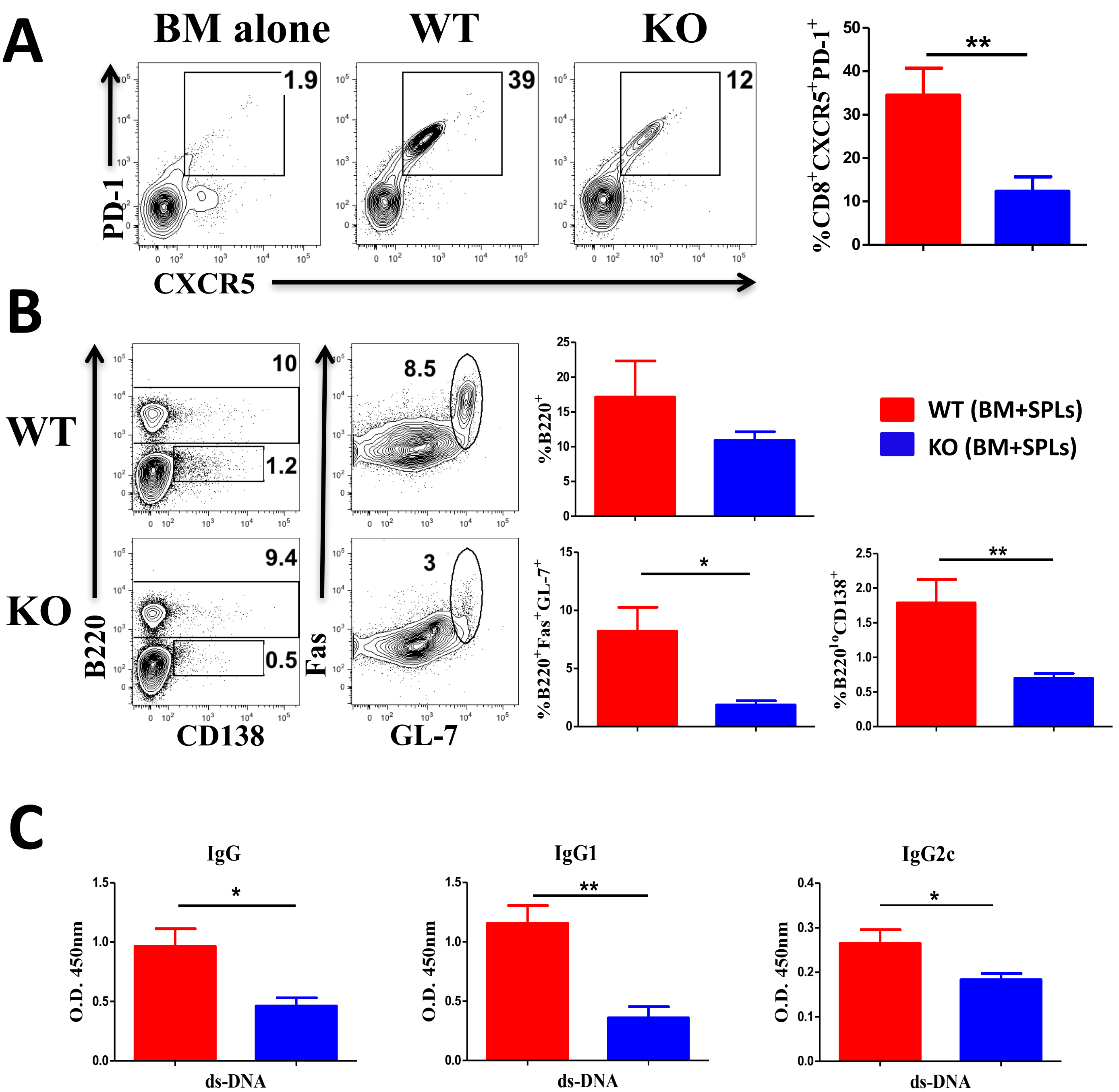

Supplemental Figure 2:

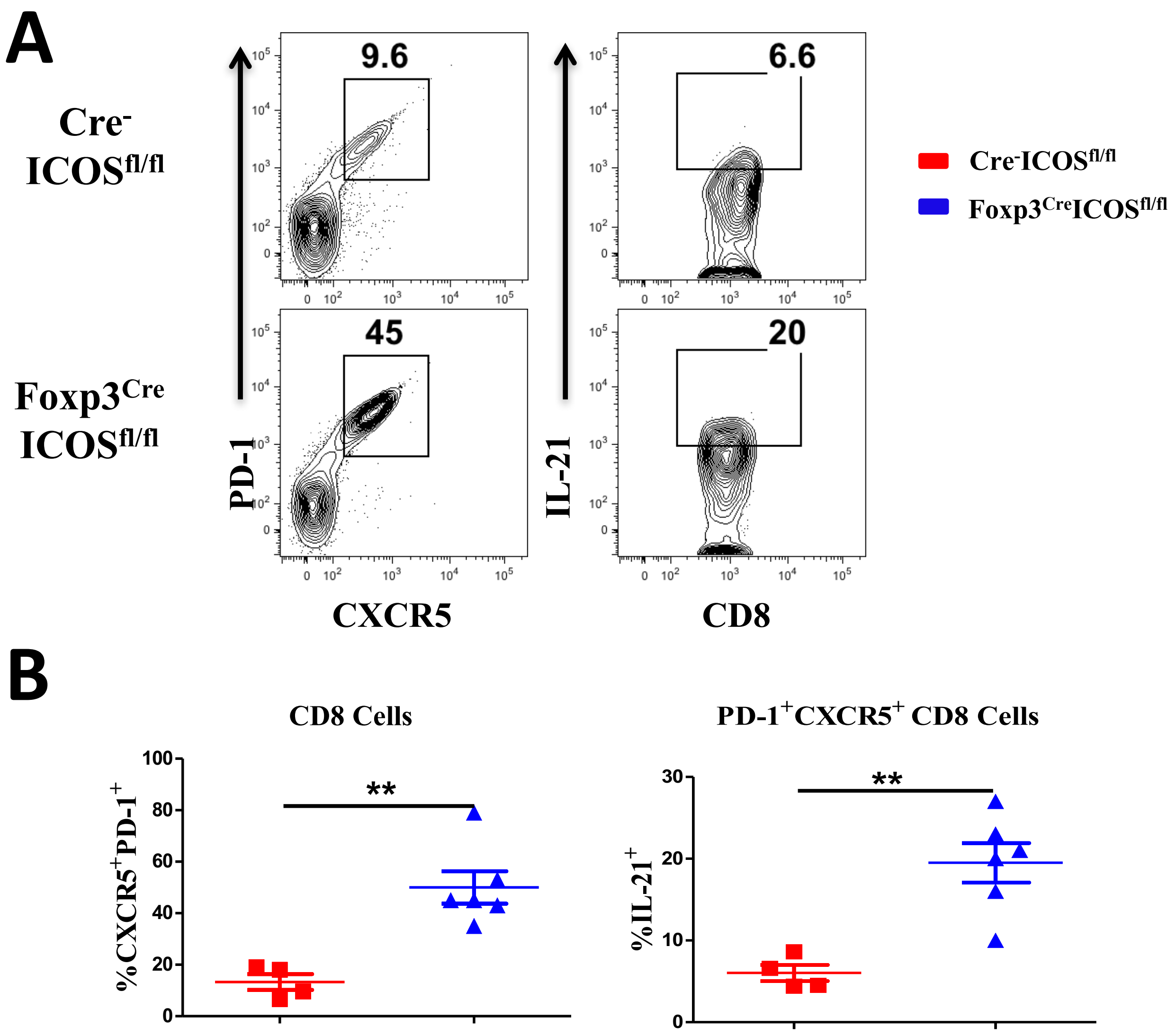

Supplemental Figure 3

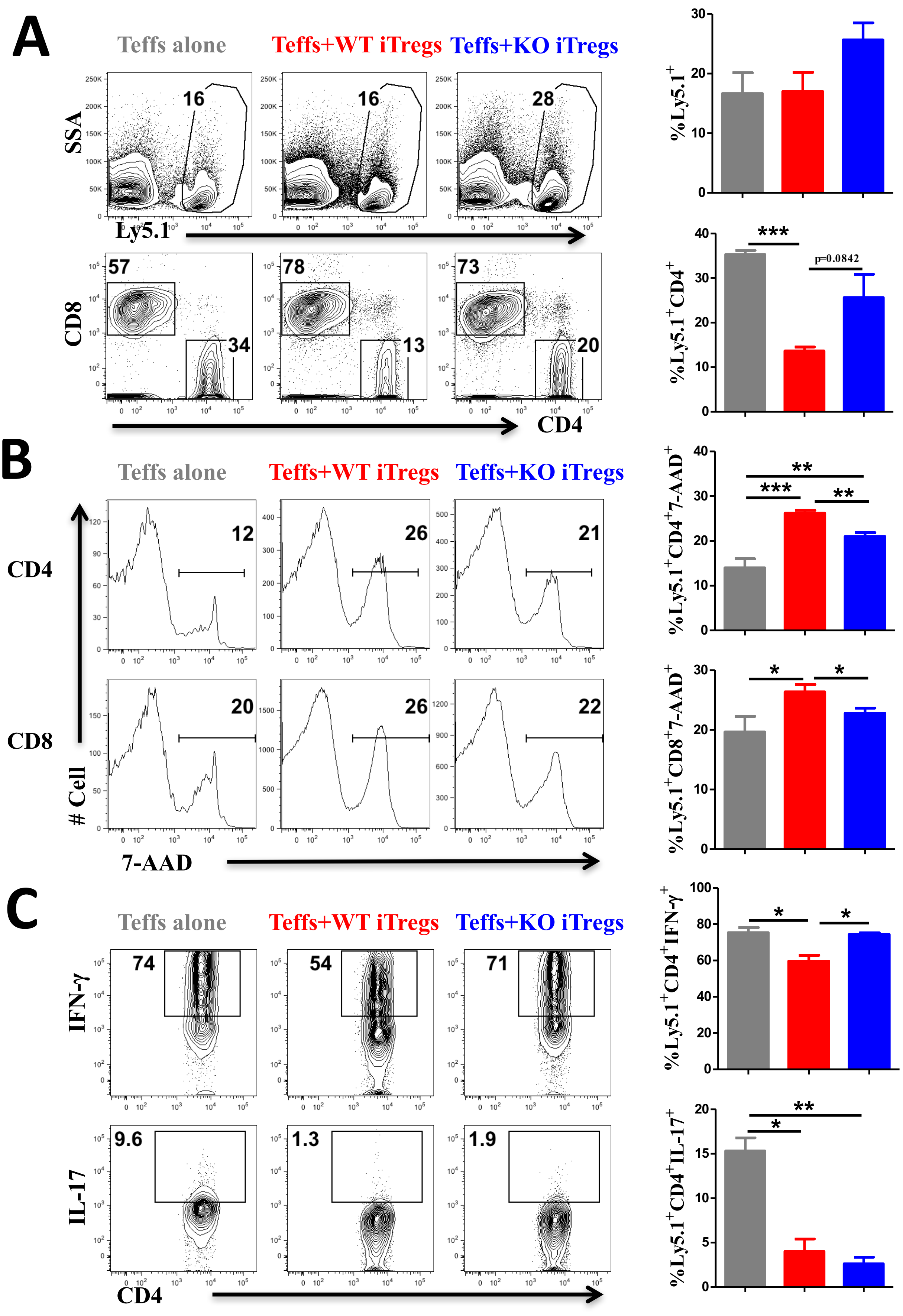

Supplemental Figure 4:

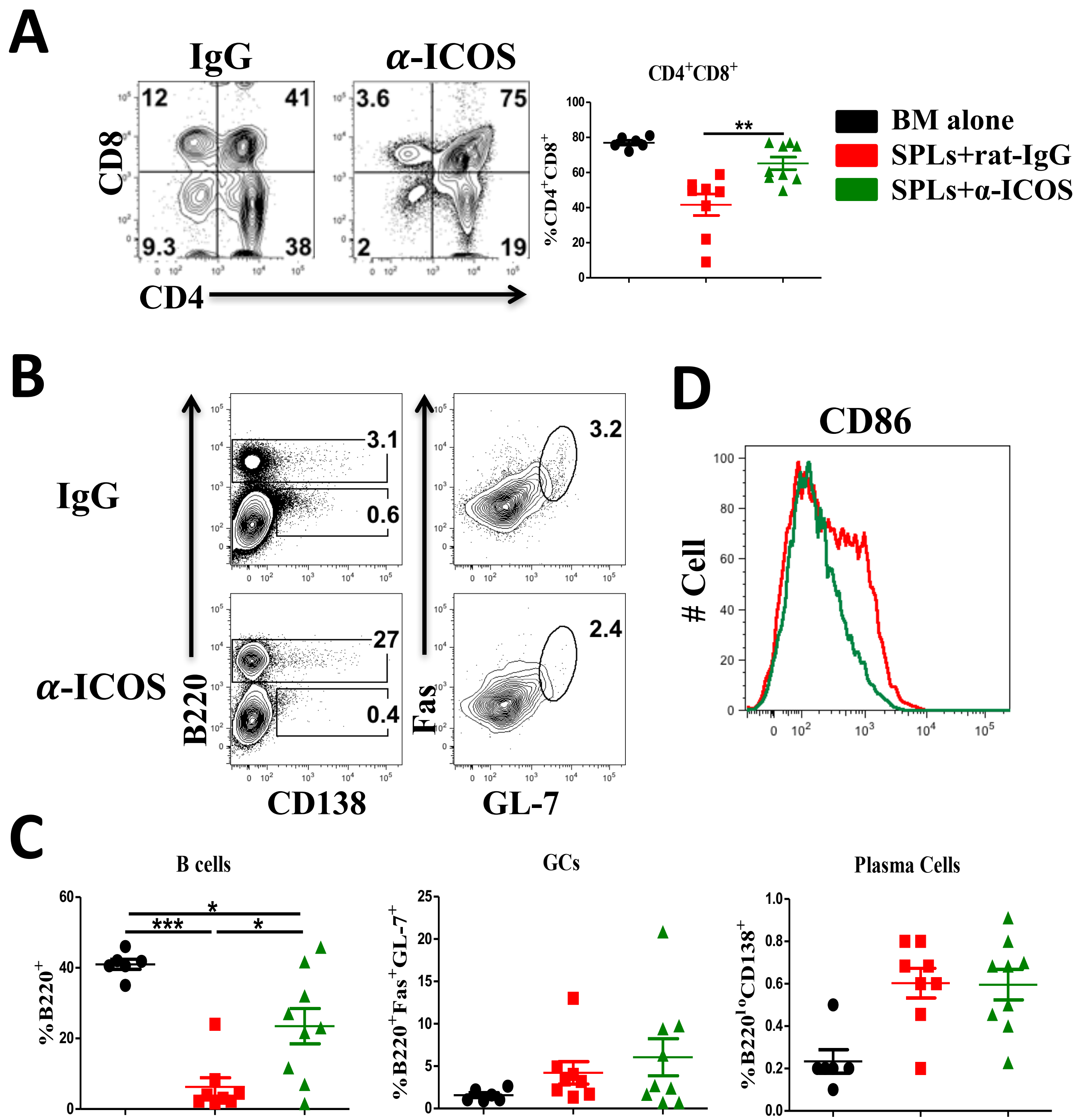

Supplemental Table 1

Subset	Impact	cGVHD outcome
**Th1**	No impact	No
**Th2**	No impact	No
**Th17**	Promote Th17 differentiation and maintenance under inflammatory condition<br>Promote IL-23R expression on Th17 cells<br>Promote Th17 differentiation and production of IL-17 and IL-21	Promote fibrosis<br>Promote Tfh differentiation<br>Promote B cell differentiation and antibody production
**Tfh**	Promote Tfh differentiation and migration into follicular areas<br>Promote Tfh cells to produce IL-21 and IL-17	Promote B cell differentiation and antibody production
**eTfh**	Promote extrafollicular Tfh differentiation<br>Promote eTfh cells to produce IL-21	Promote extrafollicular B cell differentiation and antibody production
**Treg**	Promote Tregs to inhibit T and B cell activation, differentiation and interaction<br>Inhibit fibroblast maturation and collagen secretion	Reduce pathogenicity of T and B cells
**CD8**	Promote CD8 T cell activation and proliferation<br>Promote follicular-like CD8 T cell differentiation	Promote fibrosis<br>Promote B cell differentiation and antibody production
**B cell**	Promote B cell activation, differentiation and migration through ICOS ligand signaling	Produce antibody
